# A survey of U.S. public perspectives on facial recognition technology and facial imaging data practices in health and research contexts

**DOI:** 10.1371/journal.pone.0257923

**Published:** 2021-10-14

**Authors:** Sara H. Katsanis, Peter Claes, Megan Doerr, Robert Cook-Deegan, Jessica D. Tenenbaum, Barbara J. Evans, Myoung Keun Lee, Joel Anderton, Seth M. Weinberg, Jennifer K. Wagner

**Affiliations:** 1 Mary Ann & J. Milburn Smith Child Health Outcomes, Research and Evaluation Center, Ann & Robert H. Lurie Children’s Hospital of Chicago, Chicago, Illinois, United States of America; 2 Department of Pediatrics, Feinberg School of Medicine, Northwestern University, Chicago, Illinois, United States of America; 3 Department of Electrical Engineering, ESAT/PSI, KU Leuven, Leuven, Belgium; 4 Medical Imaging Research Center, MIRC, KU Leuven, Leuven, Belgium; 5 Department of Human Genetics, KU Leuven, Leuven, Belgium; 6 Sage Bionetworks, Seattle, Washington, United States of America; 7 School for the Future of Innovation in Society, Arizona State University, Washington, District of Columbia, United States of America; 8 Department of Biostatistics & Bioinformatics, Duke University School of Medicine, Durham, North Carolina, United States of America; 9 Levin College of Law, University of Florida, Gainesville, Florida, United States of America; 10 Wertheim College of Engineering, University of Florida, Gainesville, Florida, United States of America; 11 Center for Craniofacial and Dental Genetics, Department of Oral and Craniofacial Sciences, University of Pittsburgh, Pittsburgh, Pennsylvania, United States of America; 12 School of Engineering Design, Technology, and Professional Programs, Pennsylvania State University, University Park, Pennsylvania, United States of America; National Institute of Public Finance and Policy, INDIA

## Abstract

Facial imaging and facial recognition technologies, now common in our daily lives, also are increasingly incorporated into health care processes, enabling touch-free appointment check-in, matching patients accurately, and assisting with the diagnosis of certain medical conditions. The use, sharing, and storage of facial data is expected to expand in coming years, yet little is documented about the perspectives of patients and participants regarding these uses. We developed a pair of surveys to gather public perspectives on uses of facial images and facial recognition technologies in healthcare and in health-related research in the United States. We used Qualtrics Panels to collect responses from general public respondents using two complementary and overlapping survey instruments; one focused on six types of biometrics (including facial images and DNA) and their uses in a wide range of societal contexts (including healthcare and research) and the other focused on facial imaging, facial recognition technology, and related data practices in health and research contexts specifically. We collected responses from a diverse group of 4,048 adults in the United States (2,038 and 2,010, from each survey respectively). A majority of respondents (55.5%) indicated they were equally worried about the privacy of medical records, DNA, and facial images collected for precision health research. A vignette was used to gauge willingness to participate in a hypothetical precision health study, with respondents split as willing to (39.6%), unwilling to (30.1%), and unsure about (30.3%) participating. Nearly one-quarter of respondents (24.8%) reported they would prefer to opt out of the DNA component of a study, and 22.0% reported they would prefer to opt out of both the DNA and facial imaging component of the study. Few indicated willingness to pay a fee to opt-out of the collection of their research data. Finally, respondents were offered options for ideal governance design of their data, as “open science”; “gated science”; and “closed science.” No option elicited a majority response. Our findings indicate that while a majority of research participants might be comfortable with facial images and facial recognition technologies in healthcare and health-related research, a significant fraction expressed concern for the privacy of their own face-based data, similar to the privacy concerns of DNA data and medical records. A nuanced approach to uses of face-based data in healthcare and health-related research is needed, taking into consideration storage protection plans and the contexts of use.

## Introduction

Millions of CT (computerized tomography), MRI (magnetic resonance imaging), and PET (positron emission tomography) scans are performed in the United States each year [1]. Such medical imaging has an established standard for interoperability (i.e., the Digital Imaging and Communication in Medicine or DICOM) [2]. Perhaps not surprisingly, it has been widely hoped that the inclusion of imaging would “strengthen” precision health initiatives [1]—such as the National Institutes of Health (NIH) *All of Us*^SM^ Research Program [3] and the National Cancer Institute (NCI) Cancer Moonshot^SM^ with its Imaging Data Commons [4–5]—by linking both images and image-derived data to biomedical data abstracted from electronic health records (EHRs), genetic and genomic data, patient/participant-provided information (e.g., self-reported phenotypic data), and even consumer data (i.e., data drawn from diverse online and offline sources that might yield insights into social determinants of health). Yet traditional medical imaging is the tip of the digital imaging iceberg for precision health research. Smartphones loaded with mHealth apps (enabled by Apple ResearchKit and Android ResearchStack) are now prevalent in the United States [6–9], and we can reasonably expect a surge in selfies (i.e., photos of an individual taken by that individual), posies (i.e., photos of an individual taken by another individual), and other non-DICOM (i.e., non-clinical) images—many of which would involve human faces—becoming available for precision health research [e.g., 10].

With the increased convenience of and interest in imaging for research purposes, numerous practical, ethical, legal, and social issues must be addressed. The human face is inherently identifiable—perhaps our most public personal feature. Recent work has demonstrated that under certain conditions individuals can be reidentified from seemingly anonymous MRI scan images [11]. Patients’ medical images have inadvertently appeared in Google image searches [12], and analysis of human faces can enable inferences about one’s health status [e.g., 13–20] and even one’s genomic information [21]. Understandably, many in and out of the precision health research community wonder whether the measures taken to ensure responsible stewardship of facial imaging and imaging-derived data are appropriate and adequate. For example, scholars have examined journal editorial policies and publication of medical photographs, raising concerns about privacy and confidentiality [22, 23]. Others have focused on privacy and security concerns regarding imaging without particular regard to the uniqueness of the human head and face [e.g., 24–26]. Identifiability is just one challenging ELSI (i.e., ethical, legal, and social implication) aspect of facial imaging and precision health research, however [e.g., 21 (at Suppl. Note 5), 27]. Other issues abound. Inferring health from facial appearance, for example, suffers from well-documented biases [28–30] that emerging artificial intelligence and machine learning methods have the potential both to alleviate and exacerbate.

The emergence of new facial recognition technologies (FRT) intensifies the availability of and interest in facial imaging for precision health research purposes. Several healthcare applications of FRT have been identified, including assisting in the diagnosis of certain medical conditions (such as melanoma and certain craniofacial anomalies), detecting pain and pain relief, and accurately matching patients to their medical records [16–17, 31, 32]. Other applications focus more squarely on FRT’s uses for identification and authentication, including securing access to physical spaces or computer workstations, enabling touch-free appointment check-in for patients, and even detecting or deterring healthcare fraud [33–35].

While some have warned of FRT signaling the “end of anonymity” if left unchecked [36–38], applications of FRT in healthcare settings have received limited scholarly attention. Legal scholar Seema Mohapatra has noted that current regulatory frameworks are “not well suited” to handle medical applications of FRT [39]. Bioethicist Nicole Martinez-Martin has warned that FRT applied in a healthcare setting could erode patient trust in healthcare providers and has called for research examining patient perspectives regarding use of facial recognition in healthcare settings [40]. While the perspectives of relevant professionals on ethical issues with FRT have come into clearer view following a recent international survey [41], U.S. public perspectives on FRT and their applications in healthcare and research contexts remain poorly understood. While some empirical data about public perspectives on biometrics (including facial imaging) have been published [42–44], these have not directly examined healthcare use cases or precision health research-specific aspects and also have focused on non-U.S. perspectives. One example is the public opinion survey on use of FRT in the U.K. by the Ada Lovelace Institute [45]. Moreover, other studies of data sharing in precision health research [e.g., 46] have not focused on issues specifically related to facial imaging and derived data. Therefore, to enable a better understanding of U.S. adult perspectives on facial imaging and FRT—and how these perspectives might influence trust and participation in precision health research that involves use and sharing of facial imaging, DNA, and EHR data—we conducted two online surveys: one survey focused on six types of biometrics (including facial imaging) and a wide range of societal applications and a second survey focused on facial imaging and FRT for healthcare and research applications. Our findings regarding U.S. adult public perspectives on facial imaging and FRT in non-medical contexts will be reported elsewhere. Here we report the findings of U.S. public perspectives on facial imaging and FRT specifically in healthcare and research contexts.

## Methods

We used Qualtrics Panels, a panel aggregation service provided by Qualtrics LLC (Provo, Utah, USA), to collect responses to two complementary and overlapping survey instruments. This study protocol was determined to meet exemption criteria of 45 CFR 46.104(d)(2) by the University of Pittsburgh IRB (STUDY20070193), and the study activities to be performed at Geisinger by its researchers and consultants (IRB #2020–0926) were subsequently determined not to involve “human subjects” as defined in 45 CFR 46.102.

Survey instruments were designed to incorporate new questions as well as ones adapted or inspired from published surveys [42–44]. Instruments were topically distinct, with one focused on six types of biometrics (including facial images and DNA) and their use in a wide range of societal contexts (including healthcare and research) and the other focused on facial imaging, facial recognition technology, and related data practices in health and research contexts specifically. The set of questions used in the health care and research contexts survey are provided as S1 File. An informational page explained the study, and individuals provided implicit consent to participation by proceeding beyond that informational page and responding to any questions.

Through quota sampling based on four dimensions (i.e., age, gender, race and ethnicity, and geographic region) and using eligibility criteria open to any adult residing in the U.S., we ensured our response sample would resemble the broader U.S. adult population. Anonymous survey responses were collected between November 24 and December 14, 2020. We had determined that, for simple comparisons, sample sizes for subgroups of interest as small as n = 50 would provide 80% statistical power at alpha = 0.05 to detect effect sizes of 0.23.

Statistical analyses were performed using R [47]. Survey questions eliciting nominal responses were analyzed using Pearson’s Chi-squared test. Wilcoxon sign rank tests were performed on questions eliciting ordinal responses to detect significant deviations from the sample median response. Effects of sociodemographic variables on response outcomes were tested using Kruskal-Wallis tests and post-hoc Dunn tests with Bonferroni adjustment for multiple comparisons. Associations between ordered categorical data were investigated using polychoric correlations. A statistical significance threshold of 0.05 was used. Results from the statistical tests are reported as S2 File.

## Results

A total of 4,048 adults residing in the United States responded to our two surveys, with 2,038 respondents completing the first survey focused on six different biometrics and their use in societal contexts generally and 2,010 respondents completing the survey focused on FRT in healthcare settings and matters relevant to precision health research initiatives. As shown in Table 1, the responses were elicited from a diverse group of adults in terms of age, geographic region, gender, racial and ethnic background, educational attainment, household income, and political views. Demographic factors were not found to have meaningful effects on survey responses (See S2 File).

**Table 1:** Demographics of survey respondents.

Demographic		All Respondents		Respondents to Health Research Contexts Survey Only	
		N = 4048	%	N = 2010	%
**Age**	18–25	524	12.9	261	13.0
	26–35	728	18.0	364	18.1
	36–45	678	16.7	339	16.9
	46–55	671	16.6	319	15.9
	56–65	675	16.7	338	16.8
	66–75	640	15.8	314	15.6
	76+	132	3.3	75	3.73
**Geographic Region**	South	1491	36.8	762	37.9
	West	999	24.7	479	23.8
	Midwest	840	20.8	399	19.9
	Northeast	713	17.6	368	18.3
**Gender**	Woman	2029	50.1	990	49.3
	Man	1943	48.0	978	48.7
	Non-binary	21	0.52	12	0.60
	Transgender (including Man, Transgender; Woman, Transgender; or Transgender)	13	0.32	8	0.40
**Racial and Ethnic Background**	American Indian or Alaska Native	56	1.38	39	0.02
	Asian	172	4.2	80	3.98
	Black, African American, or African	473	11.7	235	11.7
	Hispanic, Latino, or Spanish	470	11.6	235	11.7
	Native Hawaiian or other Pacific Islander	21	0.52	18	0.90
	White	2466	60.9	1184	58.9
	Black, African American, or African & Hispanic, Latino, or Spanish	9	0.22	4	0.20
	Black, African American, or African & White	52	1.28	30	1.49
	Hispanic, Latino, or Spanish & White	123	3.04	61	3.03
	Other combination of two or more categories	129	3.19	74	3.68
**Educational Attainment**	Grade 11 or below	218	5.4	105	5.22
	Grade 12 or GED	912	22.5	440	21.9
	1 to 3 years after high school	1221	30.2	581	28.9
	College 4 years or more	967	23.9	499	24.8
	Advanced degree	688	17.0	360	17.9
**Household Income**	Less than $25,000	823	20.3	400	19.9
	$25,000 - $49,999	1058	26.1	481	23.9
	$50,000 - $74,999	680	16.8	333	16.6
	$75,000 - $99,999	496	12.3	268	13.3
	$100,000 - $149,999	453	11.2	231	11.5
	$150,000 or more	368	9.1	208	10.4
**Political Views**	Conservative	1099	27.1	557	27.7
	Moderate	1568	38.7	750	37.3
	Liberal	1017	25.1	511	25.4

Totals for each demographic item do not necessarily sum to N = 4048 for all respondents or N = 2010 for health research survey respondents due to item nonresponse or selections (such as “I prefer not to answer.”) that are not displayed.

**Fig 1:**
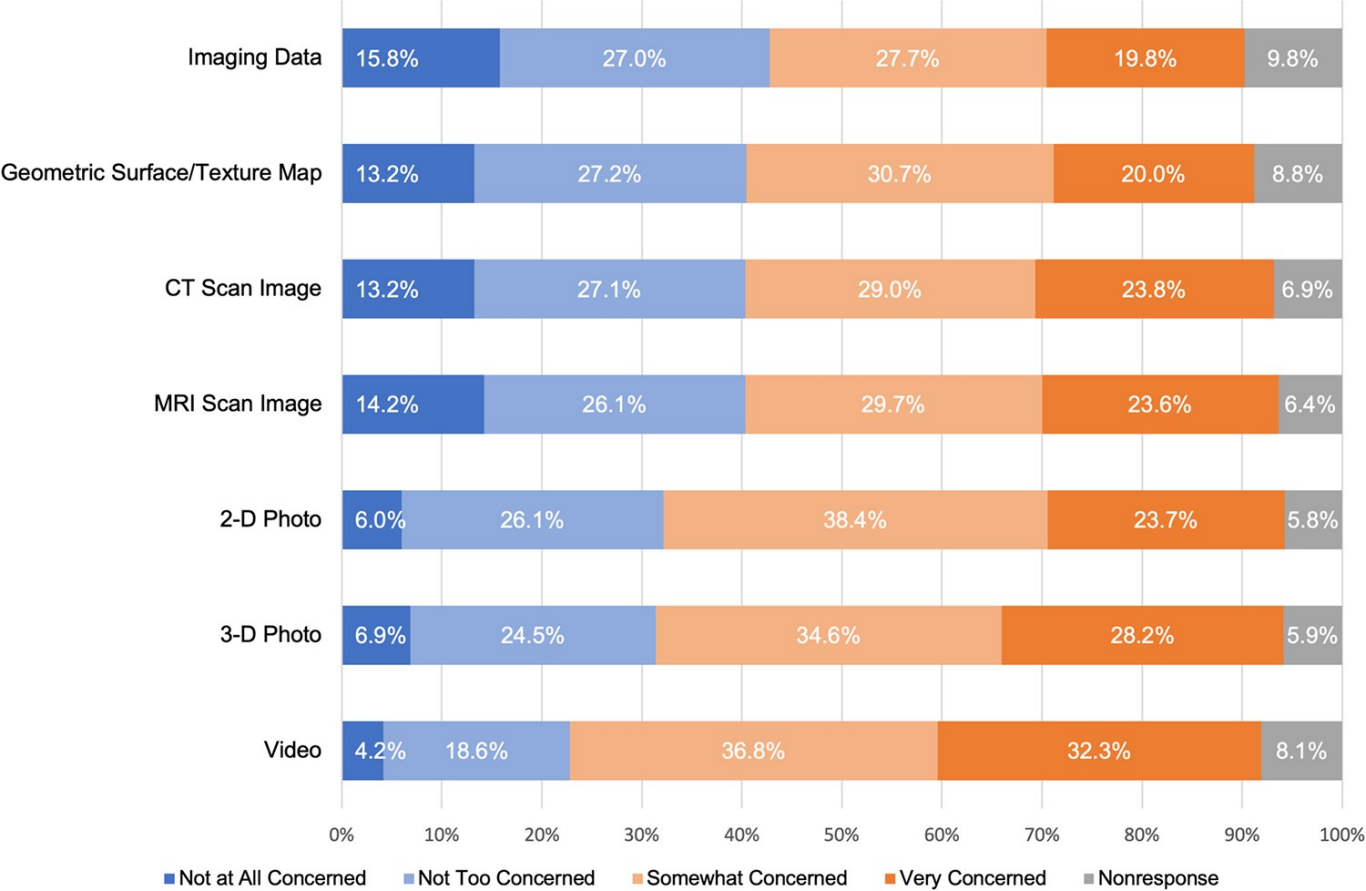
Privacy concerns regarding the use and sharing of facial images and facial imaging data in health research. Respondents were asked the following question: “There are many different types of facial imaging, and the ability to identify an individual from these different types varies. In health research, names and other identifying information are removed from facial imaging to preserve the privacy of the individual. However, reidentification is sometimes possible. Imagine that you are participating in health research involving facial images and facial imaging data. Based on what you know, how concerned are you about your privacy if the following are used and shared as part of that health research?” Proportion of respondents indicating they were very concerned (dark orange), somewhat concerned (light orange), not too concerned (light blue), and not at all concerned (dark blue) are displayed along with item nonresponses (gray).

Respondents expressed varying levels of privacy concern for a health study using and sharing facial images and facial imaging data. As shown in Fig 1, reported privacy concerns were highest for video images (71.1%, 1389/2010 noting they were “very” or “somewhat” concerned) and lowest for imaging-derived data (47.4%, 953/2010) noting they were “very” or “somewhat” concerned). Demographic factors had only small or not statistically significant effects on these privacy concerns.

**Fig 2:**
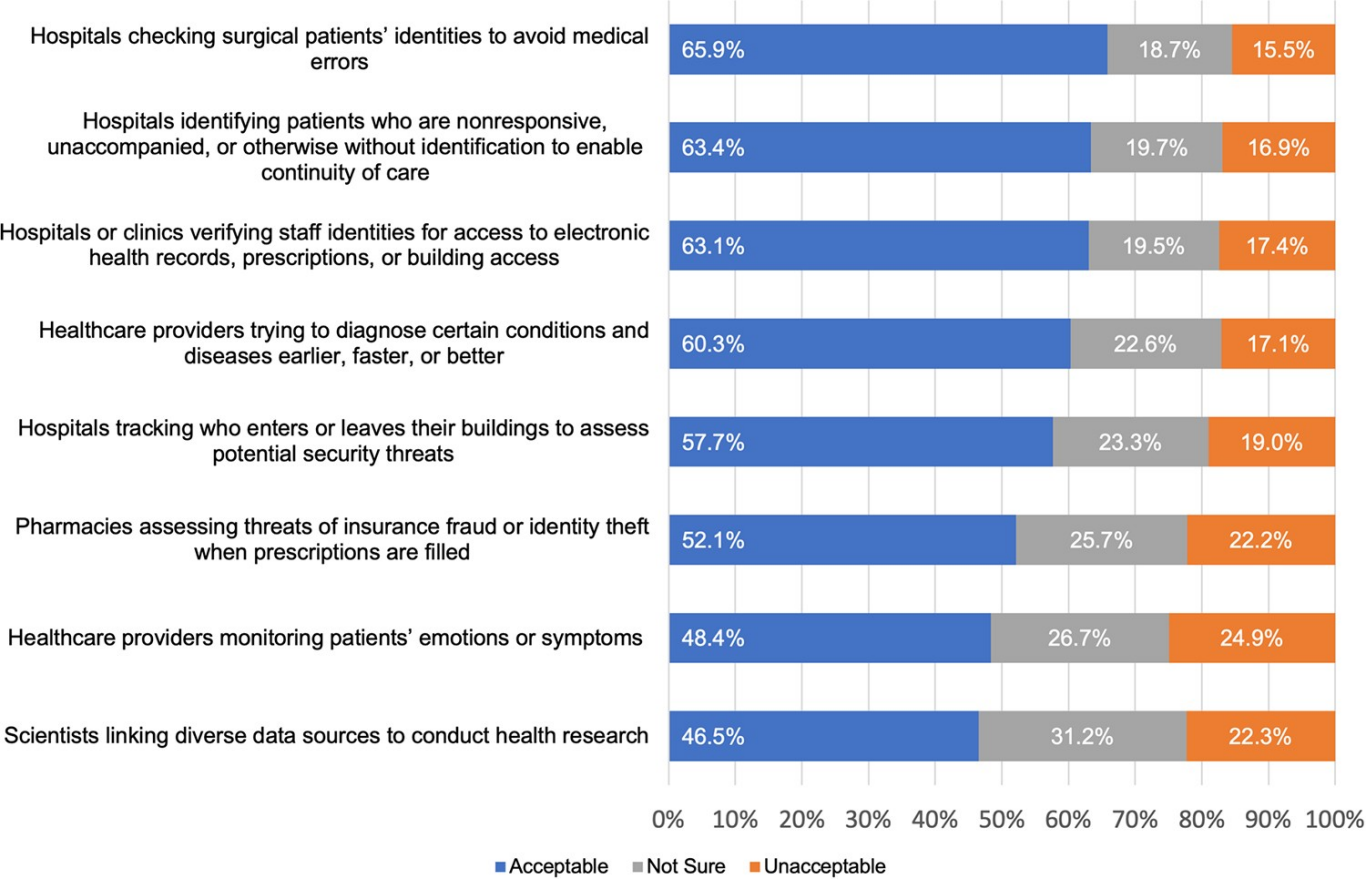
Acceptability of facial recognition technologies in eight healthcare scenarios. For each scenario, respondents reported whether the use case was acceptable (blue) or unacceptable (orange) or reported being unsure (gray) about the (un)acceptability of FRT.

Facial recognition technologies were considered acceptable by a majority of respondents in our survey in six of the eight scenarios (as shown in Fig 2). In fact, 19.8% (800/4048) of respondents considered FRT to be acceptable in all eight of the scenarios we posed, and 53.2% (2154/4048) considered it acceptable in five of the eight scenarios. The two scenarios that failed to elicit a majority acceptance were, notably, healthcare providers monitoring patients’ emotions or symptoms (48.4%, 1879/3883 reported as acceptable) and scientists linking diverse data sources to conduct health research (46.5%, 1793/3855 reported as acceptable). Demographic factors had small or no effects on acceptability of FRT in the eight scenarios.

**Fig 3:**
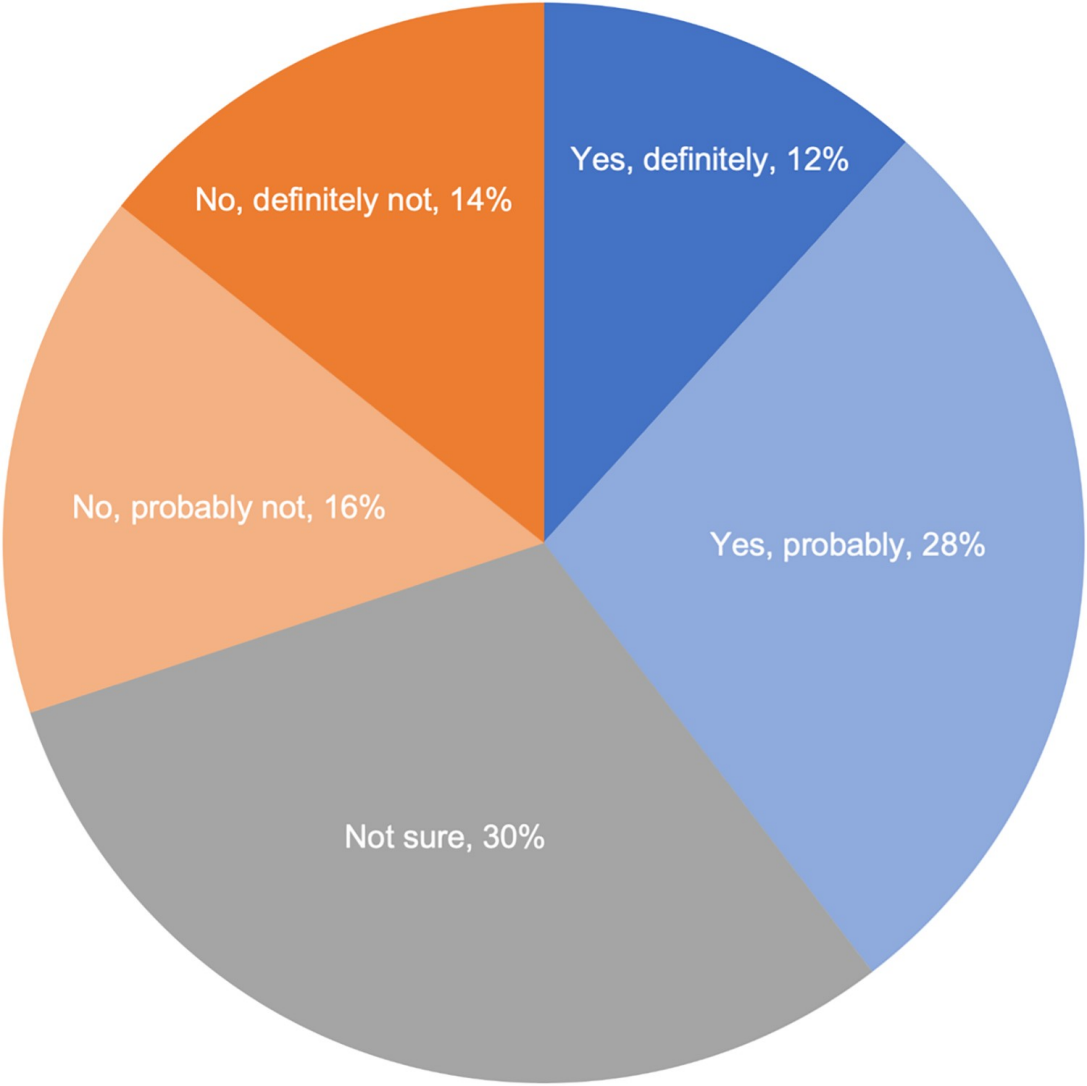
Willingness to participate in a hypothetical precision health study. Respondents were presented with this vignette: “For the next set of questions, imagine that you are asked to participate in a health study that seeks to understand a wide range of human diseases and conditions. The researchers will study your medical records (such test results and information about diseases and conditions); will collect a DNA sample to study your DNA information; and will collect images of your face to study along with any medical images and related information in your medical records. The researchers will not share study resources that could identify someone easily. Each participant will be given a unique study ID number.” Shades of blue denote those definitely (dark) and probably (light) willing to participate. Shades of orange denote those definitely (dark) and probably (light) not willing to participate. Gray shading denotes those not sure about participation.

A vignette (see S1) was used to gauge respondents’ hypothetical willingness to participate in a precision health study. The vignette explained the study seeks to understand a wide range of diseases and conditions; that the study would involve use of medical records, DNA, facial imaging, and related data; and that the researchers would take steps to prevent a participant from being easily identified (e.g., use of a study ID number). Thirty percent (30.3%, 609/2010) of respondents were not sure about participating in such as study, 30.1% (605/2010) of respondents indicated they were probably or definitely unwilling to participate, and 39.6% (796/2010) of respondents indicated they were probably or definitely willing to participate (Fig 3). Demographic factors had small or no effects on willingness to participate.

**Fig 4:**
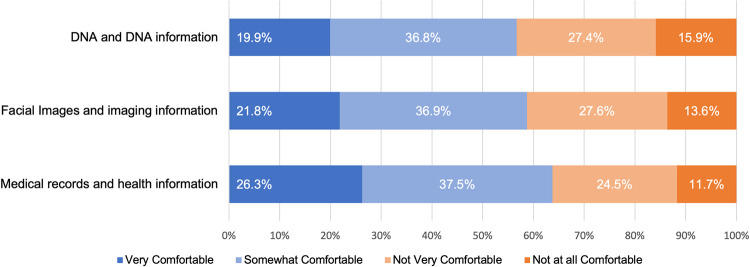
Comfort with a precision health study using medical records, facial images, DNA, and related information. Blue shading denotes the proportion of respondents who expressed they were very (dark blue) and somewhat (light blue) comfortable with a study’s use of each of the three types of research resources. Orange shading denotes the proportion of respondents who expressed they were very (dark orange) and somewhat (light orange) uncomfortable with a study’s use of each of the three types of research resources.

**Fig 5:**
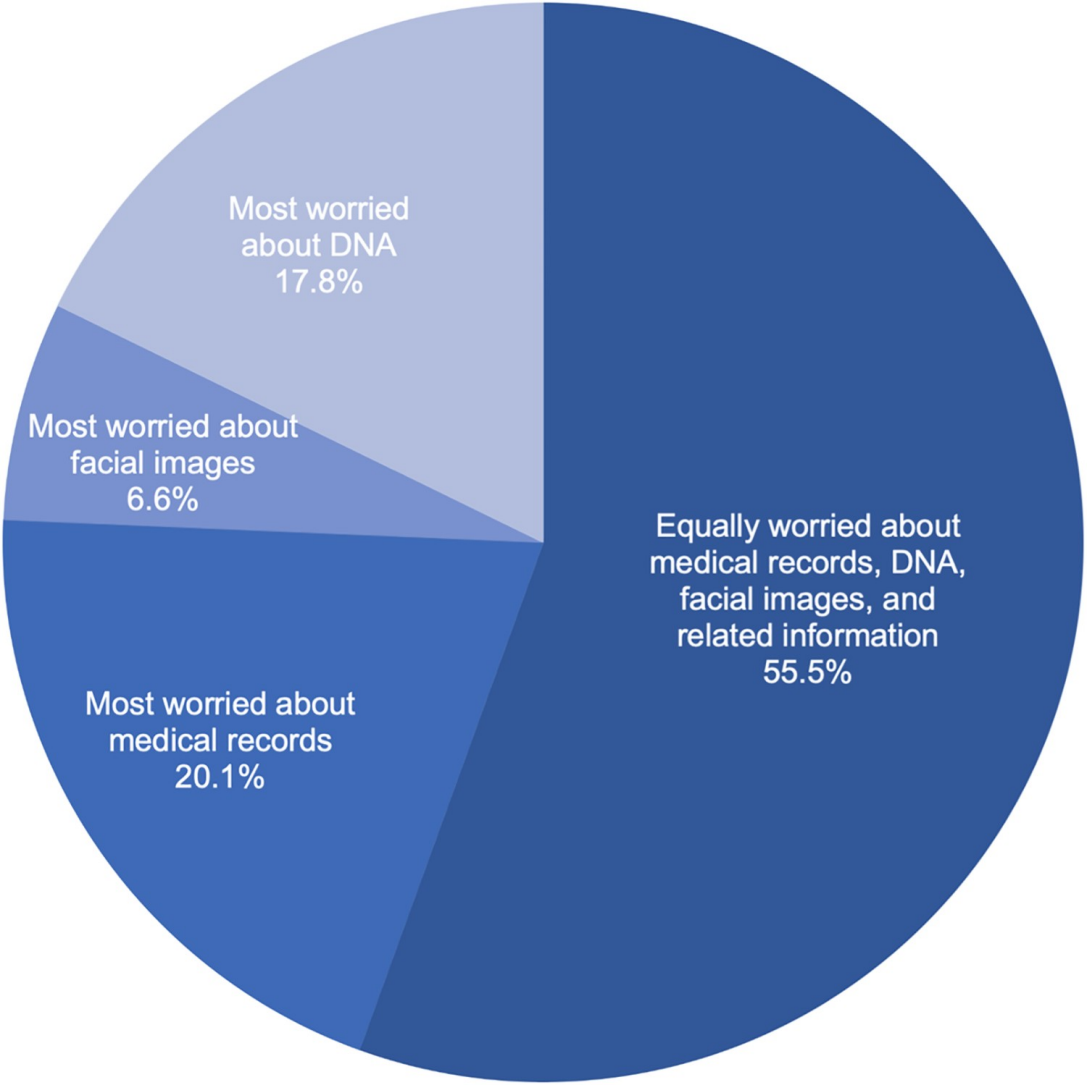
Relative privacy concerns when information is collected for research purposes. The darkest shade of blue denotes equal concern for the three types of research resources (medical records, DNA, and facial images). The second darkest shade of blue denotes those whose concerns for medical records were stronger (more) than their concerns about either facial images or DNA. The second lightest shade of blue denotes those whose concerns for facial images were stronger (more) than their concerns about either medical records or DNA. The lightest shade of blue denotes those whose concerns for DNA were stronger (more) than their concerns about either medical records or facial images.

Respondents were asked to think about their relative comfort with a precision health study using three separate types of research resources (medical records, DNA, facial images, and related information) regardless of their willingness to participate in such a study. Across our response sample (Fig 4), comfort was highest for medical records and related information followed by facial imaging and related information and then, lastly, DNA and related information (with 63.7%, 58.8%, and 56.7% reporting being very or somewhat comfortable with the research item, respectively). Because comfort could encompass respondents’ consideration of multiple unspecified factors, respondents were also asked directly about their relative privacy concerns for each research resource (Fig 5). A majority of respondents (55.5%, 1116/2010) indicated they were equally worried about the privacy of medical records, DNA, and facial images collected for precision health research. One-fifth of respondents (20.1%, 405/2010) indicated that they were more concerned about their medical records than either facial imaging or DNA. Only 6.6% (132/2010) of respondents indicated they were more concerned about facial imaging and corresponding data than other types of research resources. Prior experience with medical imaging of the head or face also was also correlated with respondents’ comfort with a health study using facial imaging and imaging data (r = 0.205, p-value = 2.01E-20). Moreover, respondents’ comfort with a health study using facial images was positively correlated with the respondents’ trust in researchers (r = 0.488, p-value = 1.24E-116) and clinicians (r = 0.429, p-value = 11.38E-87) to use facial imaging and imaging data responsibly. Similar positive correlations were found between comfort with a health study using DNA and trust in researchers (r = 0.487, p-value = 1.23E-116) and clinicians (r = 0.444, p-value = 2.61E-95) using DNA and DNA data responsibly.

**Fig 6:**
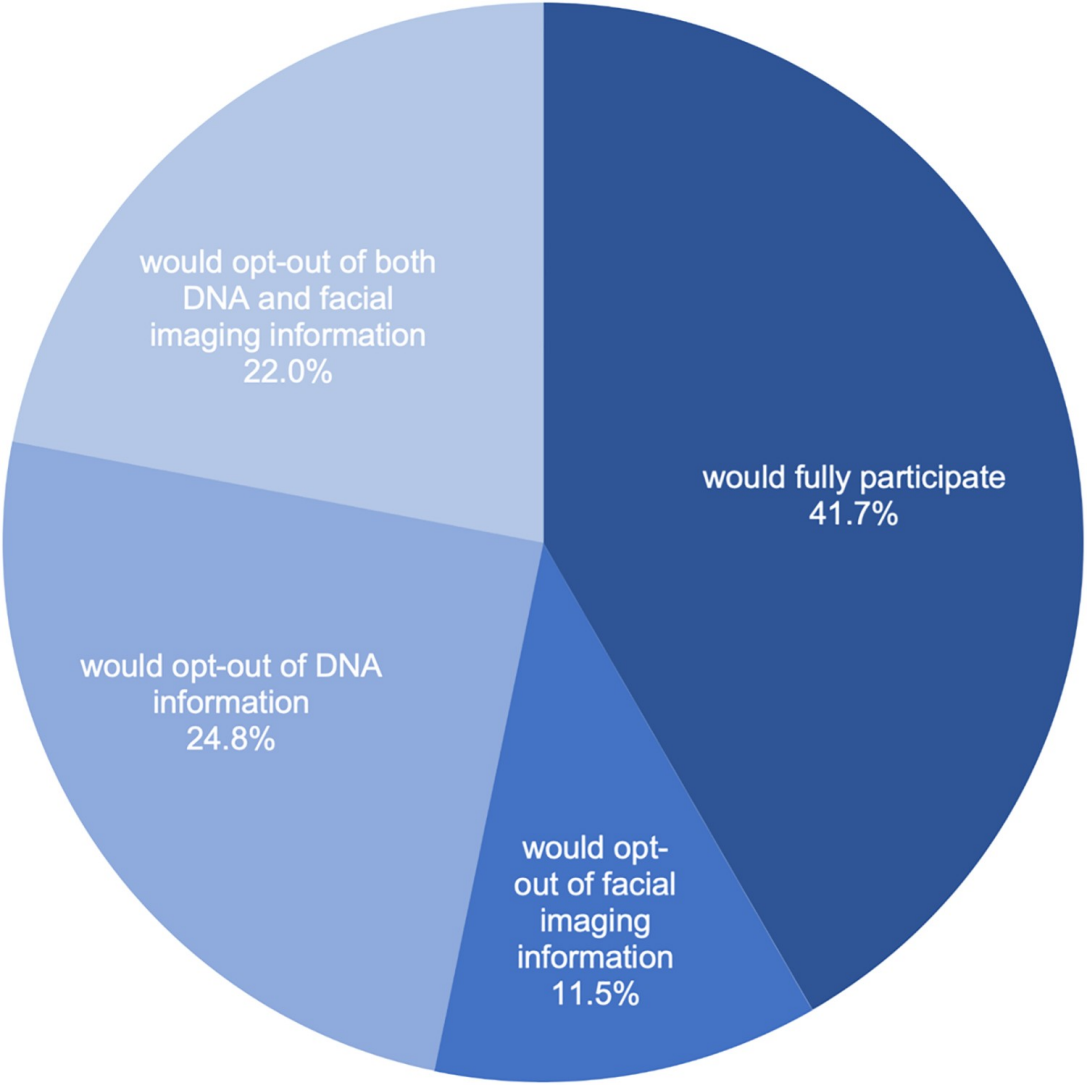
Hypothetical willingness to participate and opt-out preferences for a precision health study. The darkest shade of blue denotes willingness to participate fully including with the three types of research information (medical record, DNA, and facial imaging information). The second darkest shade of blue denotes those who would opt out of the facial imaging component. The second lightest shade of blue denotes those who would opt out of the DNA component. The lightest shade of blue denotes those who would opt out of both the DNA and facial imaging component.

When presented with information that a hypothetical study would enable participants to opt-out of the collection and use of certain research resources even though opting out could hinder the research and reduce the health discoveries that might be possible, 41.7% (838/2010) of survey respondents indicated they would participate fully. As shown in Fig 6, despite the recognition that opting out would limit the value of an individual’s data for the research, nearly one-quarter of respondents (24.8%, 498/2010) reported they would opt out of the DNA component of the study and 22.0% (442/2010) reported they would opt out of both the DNA and facial imaging component of the study. Of those respondents who expressed to opt-out of DNA, facial imaging, or both, only 29.4% (345/1172) indicated they would be willing to pay a fee to the healthcare organization conducting the study in order to process their request to opt-out of each research resource item. Demographic factors had small or no effects on opt-out preferences.

**Fig 7:**
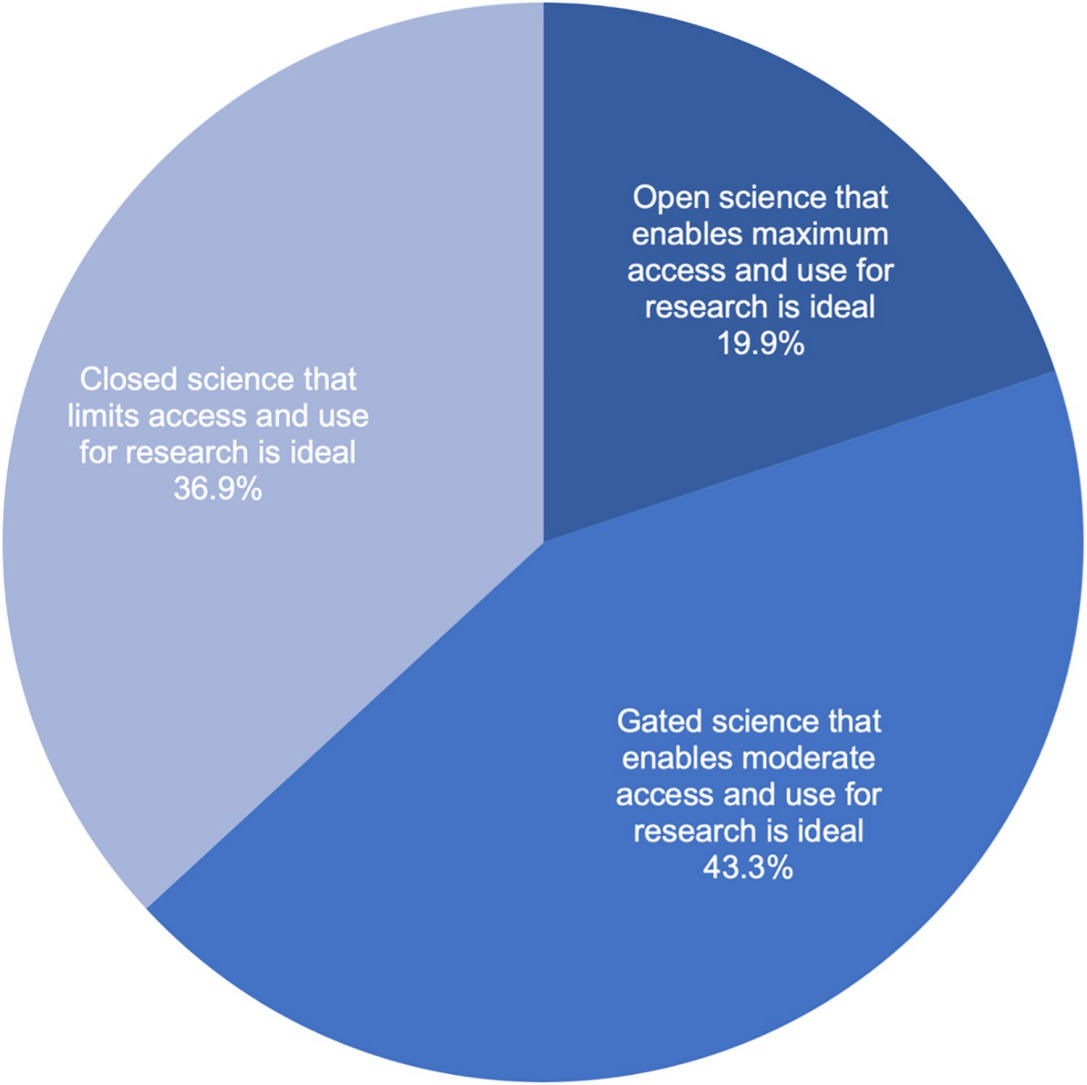
Preferred management of precision health research resources. The darkest blue shading denotes the most open of the three options, that resources should be unrestricted and made available to as many researchers as possible to answer as many research questions as possible even if that increases privacy risks to participants. The moderate blue shading denotes that research resources should be controlled and made available to qualified researchers to answer any research questions reasonably related to human health. The lightest blue shading denotes that research resources should be restricted and made available only to a few researchers and only to answer research questions stated at the beginning of the study to reduce privacy risks to participants.

Respondents were asked to consider three possible scenarios for managing precision health research resources and to select their ideal governance design: (1) one in which research resources are unrestricted and made available to as many researchers as possible and to answer as many research questions as possible to advance science even if that increases privacy risks to participants (in other words, an “open science” that enables maximum access and use); (2) one in which research resources are controlled and made available to qualified researchers and to answer research questions reasonably related to human health (in other words, a “gated science” that enables moderate access and use); and (3) one in which research resources are restricted and made available only to a few researchers and to answer only those research questions stated at the beginning of a study to reduce privacy risks to participants (in other words, a “closed science” that strictly limits access and use). No option elicited response from a majority, as shown in Fig 7. The most popular preference among this survey sample was the middle option of “gated science” (43.3%, 870/2010), and the least popular preference was the most relaxed or “open science” option (19.9%, 399/2010). Demographic factors had only small effects on these preferences. There was a statistically significant association between preference for how research resources should be managed and willingness to participate in a hypothetical precision health study (r = 0.334, p-value = 1.28E-53).

## Discussion

A better understanding of U.S. adult public perspectives on uses of facial imaging technologies and facial imaging data practices in health contexts is needed to guide healthcare industry and biomedical research decisions so that unintended consequences can be avoided. Here, we report survey findings of U.S. adult perspectives on the acceptability of eight specific health-related uses of facial recognition technologies as well as perspectives regarding relative privacy concerns regarding EHRs, facial imaging, DNA, and related data; willingness to participate in a hypothetical study involving such resources; hypothetical opt-out preferences and willingness to pay for those opt-out preferences; and general views regarding the ideal governance of research resources for precision health study purposes. While facial imaging has been an important part of specialized care (particularly pediatric genetics) for decades, biomedicine is changing–becoming much more anticipatory, occurring in digital spaces, involving larger and larger datasets containing data assets repurposed from diverse sources, and introducing new risks. While the U.S. public seems to have fairly high levels of trust in healthcare providers and researchers with use of facial imaging and FRT, we found that people are not necessarily fully supportive of expanded uses in healthcare settings and might even be uneasy about them. How healthcare professionals choose to proceed could have substantial impacts on levels of public trust in the profession generally [48].

Our findings have implications for the implementation of FRT in healthcare settings. Six of the eight use cases were considered acceptable uses of facial recognition technology by a majority of respondents. However, even the use case that elicited the most favorable response (using facial recognition for patient identification to avoid medical errors) was only considered acceptable by 65.9%. Notably, the two scenarios that did not elicit favorable views by a majority were (1) monitoring patients’ emotions or symptoms (considered acceptable by 48.4%) and (2) linking diverse data sources to conduct health research (considered acceptable by 46.5%). Additional research would be useful to understand what factors are contributing to the perspectives of those who expressed they were unsure about these two uses (26.7% and 31.2%, respectively) or expressed these two uses were unacceptable (24.9% and 22.3%, respectively). Our constructed scenarios notably did not include potentially relevant information to assess alternatives to FRT, relative financial costs involved, or comparative effectiveness. Before healthcare systems advance plans to implement facial recognition technologies, it would be useful to conduct an in-depth examination of these and other factors, including (1) aspects related to distrust of technology partners that might be involved; (2) mitigation strategies deployed to minimize risks of data breaches or leaks; and (3) widely varying local ordinance and state law restrictions on biometrics generally and facial recognition specifically.

Our findings also have implications for data management and sharing policies, a matter of critical importance with the anticipated implementation of the updated NIH Policy for Data Management and Sharing [49] and the ongoing development of responsible data practices for the *All of Us*^SM^ Research Program [3], NCI Imaging Data Commons [4, 5], and imaging science that might be pursued via other precision health initiatives (such as the Geisinger MyCode® Community Health Initiative) [50]. Here, we observed levels of comfort with a study using facial imaging and related imaging data that fell between those levels for a study using EHR data and genomic data. When asked about relative privacy concerns specifically (as opposed to other factors that could affect perceived comfort), only 6.6% of respondents indicated they were most worried about facial images. Interestingly, of those who indicated they did not worry equally about medical records, DNA, facial imaging, and related data, the most common perspective was that they were most concerned about their medical records (not their DNA or facial images). A limitation of our study, however, is that we did not explore the possibility that individuals might prefer to opt-out of an EHR component of a hypothetical precision health study while still being willing to participate in DNA and facial imaging components. These findings suggest that, at least from U.S. public perspectives, responsible stewardship of facial imaging and imaging-derived data need not be heightened compared to the approaches taken with EHR or genomic data. Notably, however, our findings regarding willingness to participate and attitudes toward biometrics and data sharing suggest that the actual participants of precision health studies might be biased toward those individuals who are more accepting of biometrics than non-participants. Such sampling biases need to be recognized and addressed by designers of precision health initiatives upstream of proposed data use and upstream of recruitment, as individuals skeptical or critical of biometrics and broad data sharing nevertheless comprise a sizable portion of users served by health services. Additional research is needed to understand the needs, interests, and concerns of those critical of FRT and other technological advancements (i.e., “biodefectors” engaged in “informed refusals” [51])—particularly if equitable precision health practices and data justice are to be prioritized and realized. Moreover, that we did not find demographic factors (notably gender or racial and ethnic background) to have meaningful effects on perspectives on facial imaging technologies and imaging data practices in health-related contexts is worthy of further research given the known biases of FRT and documented controversies regarding FRT in social contexts outside of the health-related applications we explored here [e.g., 52–55]. Qualitative research is needed to interpret these preliminary findings.

It is convenient to distinguish the use of facial imaging directly for medical or precision health research purposes on the one hand from use of facial imaging and FRT for non-medical purposes on the other. The appropriateness of such a conceptual distinction should not be assumed without critical examination, however. Policy and regulatory frameworks often focus on the actor (as opposed to intentions or acts and omissions). HIPAA is an example of this, where the statutory requirements apply to certain actors (i.e., covered entities and, by contractual extension, business associates). Multiple use cases for FRT exist in healthcare settings, and the boundaries might not be clearly drawn regarding how a healthcare organization—not to mention individual clinicians, researchers, IRBs, security officers, administrators, executives, and others employed there—accesses and uses the diverse facial imaging resources in the organization’s possession or within its reach. Careful deliberation, interdisciplinary decision-making, and continuous evaluation are critical to ensure actors manage facial images and imaging data responsibly and deploy FRT cautiously with the ethical, legal, and social implications of doing so in mind. Purpose creep and dataveillance risks within healthcare settings warrant additional empirical and normative research.

The recent picture archiving and communication system (PACS) guidance for the healthcare industry issued by the National Institute of Standards and Technology (NIST) [56] contains nearly 400 pages of important technical information, describing PACS as the “authoritative repository of medical image information” and recognizing that it “cannot operate in isolation” (p. 1). Nevertheless, this guidance offers no mention of how its intended audience (biomedical and cybersecurity engineers, healthcare technology managers, and support staff) would or should effectively collaborate with Institutional Review Boards or others to ensure proper safeguards when trying to use images in and out of PACS for research and non-medical purposes. ELSI research is needed to understand how facial images and imaging data are being managed and used in practice and how familiar research professionals are with the privacy and cybersecurity aspects of their work. Additional ELSI research is also needed to examine whether and how a “HIPAA knowledge gap” (i.e., the gap between what individuals think HIPAA allows or disallows and what HIPAA actually allows with their data) or limited data literacy (e.g., comprehension of concepts such as “de-identified data”) influences trust in healthcare organizations and professionals and support of or opposition to precision health initiatives, healthcare applications of FRT, and policy approaches.

## Supporting information

S1 File Health care and research contexts survey questions. The full set of questions for the health care and research contexts survey that were programmed into Qualtrics are provided.
(DOCX)

S2 File Statistical test results. The statistical test results performed on the survey responses are displayed.
(XLSX)

## Abbreviations

CT: computerized tomography
EHR: electronic health record
ELSI: ethical, legal, and social implications
FRT: facial recognition technology/ies
MRI: magnetic resonance imaging
NCI: National Cancer Institute
NIH: National Institutes of Health
NIST: National Institute of Standards and Technology
PACS: picture archiving and communication system
PET: positron emission tomography
