# Correction: A survey of U.S. public perspectives on facial recognition technology and facial imaging data practices in health and research contexts

**DOI:** 10.1371/journal.pone.0261738

**Published:** 2021-12-16

**Authors:** The PLOS ONE Staff

The Competing Interests statement published incorrectly. The publisher apologizes for the error. The correct statement is as follows: The authors have read the journal’s policy and the authors of this manuscript have the following competing interests: JT discloses a consulting relationship with Genetika, and JKW discloses paid employment with the Center for Translational Bioethics and Health Care Policy at Geisinger during the conduct of this study but no active competing interests at the time of publication. The authors have declared that no other competing interests exist and that the authors have no financial interests (e.g., patents, products in development, or marketing products) related to this study to declare. This does not alter our adherence to PLOS ONE policies on sharing data and materials.
