# Supplementary material for: A survey of U.S. public perspectives on facial recognition technology and facial imaging data practices in health and research contexts
Source: PLoS One. 2021 Oct 14;16(10):e0257923. doi: 10.1371/journal.pone.0257923 (PMC8516205; doi:10.1371/journal.pone.0257923)
Supplement: S1 File — The full set of questions for the health care and research contexts survey that were programmed into Qualtrics are provided. (DOCX) [file pone.0257923.s001.docx]

**S1 File. Health Care and Research Contexts Survey Questions**

**References for Question Design:**

Previous published works (including the *All of Us*^SM^ Research Program, PEW Research Center 2019, Garman and Barber 2018, Kugler 2019, and Sanderson, et al. 2017) were used for inspiration for this survey instrument, and several questions deployed similar or identical phrasing and format. Questions 1-6 were modeled closely on those used for the *All of Us^SM^* Research Program (available at https://www.researchallofus.org/survey-explorer/) but some answer options were streamlined for quicker administration. Questions 7 and 8 were inspired by similar questions used by PEW Research Center 2019 and reported by Garman and Barber 2018. Question 11 was modeled on survey results reported by Garman and Barber 2018 but DNA was added and two other biometric modalities were removed. Question 12 was modeled on survey results reported Garman and Barber 2018 to include DNA. Question 16 was adapted from PEW Research Center 2019 to explore trust in additional social actors beyond response items (a)-(c), and Question 17 was modeled to explore parallel perspectives for DNA. Question 19 was adapted from PEW Research Center 2019 to explore acceptability of facial recognition technologies in eight new contexts. Question 20 was adapted from PEW Research Center 2019 to explore perceived effectiveness in two additional tasks displayed as response items (d) and (e). Question 22 was drawn from Sanderson et al. 2017. Questions 25-26 were adapted from Kugler 2019. Question 27 was inspired by Sanderson et al. 2017. Questions 1-20 were also used in the societal contexts survey instrument.

**Basic Questions About You**

1. In which age group do you belong?
   1. 18 to 25 years old
   2. 26 to 35 years old
   3. 36 to 45 years old
   4. 46 to 55 years old
   5. 56 to 65 years old
   6. 66 to 75 years old
   7. 76 years and older
2. Where in the United States do you currently live? [pull-down menu for 50 states, district, and 5 US territories]
3. Which categories describe you? Select all that apply. Note, you may select more than one group.
   1. American Indian or Alaska Native
   2. Asian
   3. Black, African American, or African
   4. Hispanic, Latino, or Spanish
   5. Middle Eastern or North African
   6. Native Hawaiian or other Pacific Islander
   7. White
   8. None of these fully describe me. I identify as: (free text)
   9. I prefer not to answer.
4. What terms best express how you describe your gender identity? Select all that apply.
   1. Man
   2. Woman
   3. Non-binary
   4. Transgender
   5. None of these describe me. I identify as: (free text)
   6. I prefer not to answer.
5. What is the highest grade or year of school you completed?
   1. Never attended school or only attended kindergarten
   2. Grades 1 through 4 (Primary school)
   3. Grades 5 through 8 (Middle school)
   4. Grades 9 through 11 (Some high school)
   5. Grade 12 or GED (High school graduate)
   6. 1 to 3 years after high school (Some college, Associate’s degree, or technical school)
   7. College 4 years or more (College graduate)
   8. Advanced degree (Master’s, Doctorate, etc.)
   9. I prefer not to answer.
6. What is your annual household income from all sources?
   1. Less than $25,000
   2. $25,000 - $49,999
   3. $50,000 - $74,999
   4. $75,000 - $99,999
   5. $100,000 - $149,999
   6. $150,000 or more
   7. I prefer not to answer.
7. Which most closely describes your political views today?
   1. Democrat
   2. Independent but leaning Democrat
   3. Independent but leaning Republican
   4. Republican
   5. None of these fully describe me.
   6. I prefer not to answer.
8. Which most closely describes your political views in general today?
   1. Liberal
   2. Moderate
   3. Conservative
   4. I prefer not to answer.

**Your Relevant Experiences**

1. Have you ever provided a DNA sample for any reason?
2. Yes
3. No
4. Not sure
5. Have you ever had medical imaging of your head or face—such as an MRI scan (magnetic resonance imaging scan), CT scan (computed tomography scan), or medical photographs of your head or face?
6. Yes
7. No
8. Not sure
9. Indicate your experience with each of these six types of biometrics. For example, perhaps you have used biometrics (a fingerprint or face scan) to unlock your smartphone or computer; perhaps you used biometrics (such as a palm scan) to enter a building with restricted access. Please think about all possible contexts.

| Yes, I have experienced this type of biometric. | No, I have not experienced this type of biometric. | I am not sure if I have experienced this type of biometric. |
| --- | --- | --- |

1. Fingerprint (digital or ink)
2. Voice sample
3. Facial image
4. Eye (iris/retina) scan
5. Hand geometry (palm) scan
6. DNA (genetic profile or fingerprint)

**Your Perspectives About Biometrics**

1. Rank the types of biometrics in the order from the type you are most comfortable providing (1) to the type you are least comfortable providing (6) to an organization. [options randomized]
2. Fingerprint
3. Facial image
4. Voice sample
5. Eye (iris/retinal) scan
6. Hand geometry (palm) scan
7. DNA (genetic profile or fingerprint)
8. How does your opinion of biometrics today compare with your opinion of biometrics five years ago?
9. My opinion of biometrics is **MORE** favorable today than it was five years ago.
10. My opinion of biometrics has **NOT CHANGED** over the past five years.
11. My opinion of biometrics is **LESS** favorable than it was five years ago.
12. Which statement best describes how the COVID-19 pandemic has affected your opinion of the use of biometrics in society?
    1. The pandemic has made me **MORE** comfortable with use of biometrics in society.
    2. The pandemic has **NOT CHANGED** my opinion regarding the use of biometrics in society.
    3. The pandemic has made me **LESS** comfortable with use of biometrics in society.

**Your Perspectives on Related Issues**

1. How concerned are you about the potential misuses of your personal information in general?
2. Very concerned
3. Somewhat concerned
4. Not very concerned
5. Not concerned at all
6. Don’t know
7. How much, if at all, do you trust the following groups to use **facial recognition technology** responsibly?

| A great deal | Somewhat | Not too much | Not at all | No answer |
| --- | --- | --- | --- | --- |

1. Advertisers
2. Technology companies
3. Law enforcement agencies
4. Intelligence agencies
5. Health researchers/scientists
6. Healthcare providers/clinicians
7. Employers
8. Schools/Universities
9. Retailers
10. State Government
11. Federal Government
12. Foreign Government
13. How much, if at all, do you trust the following groups to use **DNA and DNA data** responsibly?

| A great deal | Somewhat | Not too much | Not at all | No answer |
| --- | --- | --- | --- | --- |

1. Advertisers
2. Technology companies
3. Law enforcement agencies
4. Intelligence agencies
5. Health researchers/scientists
6. Healthcare providers/clinicians
7. Employers
8. Schools/Universities
9. Retailers
10. State Government
11. Federal Government
12. Foreign Government
13. In your opinion, are biometric data protection and privacy laws adequate in the United States?
14. Yes
15. No
16. Not sure
17. In your opinion, is it acceptable or unacceptable to use facial recognition technology in the following situations? [options randomized]

| Acceptable | Unacceptable | Not Sure | No Answer |
| --- | --- | --- | --- |

1. Hospitals checking surgical patients’ identities to avoid medical errors.
2. Hospitals tracking who enters or leaves their buildings to assess potential security threats.
3. Hospitals identifying patients who are nonresponsive, unaccompanied, or otherwise without identification to enable continuity of care.
4. Healthcare providers trying to diagnose certain conditions and diseases earlier, faster, or better.
5. Healthcare providers monitoring patient’s emotions or symptoms (such as pain, fear, relief, anger, mistrust, sadness, happiness, or satisfaction).
6. Pharmacies assessing threats of insurance fraud or identity theft when prescriptions are filled.
7. Hospitals or clinics verifying staff identities for access to electronic health records, prescriptions, or building access.
8. Scientists linking diverse data sources to conduct health research.

**Your Understanding of Facial Imaging Technology**

1. Based on what you know, how effective do you think facial recognition technology is at the following tasks?

| Very  effective | Somewhat effective | Not too effective | Not effective  at all | No answer |
| --- | --- | --- | --- | --- |

- 1. Accurately identifying individual people
  2. Accurately assessing someone’s sex or gender
  3. Accurately assessing someone’s race or ethnicity
  4. Accurately detecting someone’s emotions or feelings
  5. Accurately diagnosing someone’s medical conditions

1. There are many different types of facial imaging, and the ability to identify an individual from these different types varies. In health research, names and other identifying information are removed from facial imaging to preserve the privacy of the individual. However, reidentification is sometimes possible. Imagine that you are participating in health research involving facial images and facial imaging data. Based on what you know, how concerned are you about your privacy if the following are used and shared as part of that health research?

|  | Very concerned | Somewhat concerned | Not too concerned | Not at all concerned | Not Sure |
| --- | --- | --- | --- | --- | --- |
| 1. Video |  |  |  |  |  |
| 1. 2-Dimensional photo |  |  |  |  |  |
| 1. 3-Dimensional photo |  |  |  |  |  |
| 1. MRI scan image |  |  |  |  |  |
| 1. CT scan image |  |  |  |  |  |
| 1. Geometric surface/Texture map |  |  |  |  |  |
| 1. Imaging data (that is, measurements but not the images themselves) |  |  |  |  |  |

For the next set of questions, imagine that you are asked to participate in a health study that seeks to understand a wide range of human diseases and conditions. The researchers will study your medical records (such test results and information about diseases and conditions); will collect a DNA sample to study your DNA information; and will collect images of your face to study along with any medical images and related information in your medical records. The researchers will not share study resources that could identify someone easily. Each participant will be given a unique study ID number.

1. If you were asked, would you take part in the study?
2. No, definitely not
3. No, probably not
4. Not sure
5. Yes, probably
6. Yes, definitely
7. Regardless of how you answered the previous question, how comfortable would you be with the health study using your medical records, DNA, and facial images?

|  | Very comfortable | Somewhat Comfortable | Not very comfortable | Not at all comfortable |
| --- | --- | --- | --- | --- |
| 1. Medical records and health information |  |  |  |  |
| 1. DNA sample and DNA information |  |  |  |  |
| 1. Facial images and imaging information |  |  |  |  |

1. Which of the following statements best reflects the privacy concerns you have for your medical records, DNA samples, facial images, and related information when collected for research purposes?
   1. I am EQUALLY worried about the privacy of my medical records, DNA, facial images, and related information.
   2. I am MORE worried about the privacy of my MEDICAL RECORDS than I am my DNA, facial images, and related information.
   3. I am MORE worried about the privacy of my DNA than I am my medical records, facial images, and related information.
   4. I am MORE worried about the privacy of my FACIAL IMAGES than I am my medical records, DNA, and related information.
2. Now imagine that this study allowed participants to opt-out of sharing certain types of materials and information, even though opting out will hinder research and reduce the health discoveries that could be possible. Which statement best reflects how you would participate or opt-out?
3. I would participate fully and allow all of my health information, DNA information, and facial imaging information to be used for the research.
4. I would opt-out of DNA information even though that will limit the value of my data for research.
5. I would opt-out of facial imaging information, even though that will limit the value of my data for research.
6. I would opt-out of both DNA information and facial imaging information, even though that will limit the value of my data for research.
7. [Skip Logic 🡪 For those who answered the previous question with b, c, or d] Imagine the organization conducting the study can only fulfill opt-out requests if it charges individuals a one-time fee for each opt-out item. Would you be willing to pay some amount of money for the organization to process your opt-out request?
8. Yes
9. No
10. Which of the following statements best describes your opinion of how access to research resources (medical records, health data, DNA samples, DNA data, facial images, and facial imaging data) should be managed?
    1. Research resources should be UNRESTRICTED and made available to AS MANY RESEARCHERS AS POSSIBLE and to answer AS MANY RESEARCH QUESTIONS AS POSSIBLE to advance scientific discoveries even if that increases privacy risks to participants. Participants should only be asked once for general permission. In other words, open science that enables maximum access and use for research is ideal.
    2. Research resources should be CONTROLLED and made available to RESEARCHERS WHO ARE QUALIFIED and to answer ANY RESEARCH QUESTIONS REASONABLY RELATED TO HUMAN HEALTH. Participants should only be asked once for general permission but should be able to see which researchers have access and what research questions have been studied. In other words, gated science that enables moderate access and use for research is ideal.
    3. Research resources should be RESTRICTED and made available only to A FEW RESEARCHERS and to answer ONLY THE RESEARCH QUESTIONS STATED AT THE BEGINNING OF THE STUDY to reduce privacy risks to participants. Participants should be asked for specific permission each time new researchers want access to the research resources the individual contributed and each time new research questions are to be studied. In other words, closed science that limits access and use for research is ideal.
